# Rifaximin for small intestinal bacterial overgrowth in patients without irritable bowel syndrome

**DOI:** 10.1186/s12941-014-0049-x

**Published:** 2014-10-17

**Authors:** Doron Boltin, Tsachi Tsadok Perets, Einav Shporn, Shoshana Aizic, Sigal Levy, Yaron Niv, Ram Dickman

**Affiliations:** Gastroenterology Laboratory, Rabin Medical Center, Beilinson Campus, 39 Jabotinski Street, Tel Aviv, 49100 Israel; The Neurogastroenterology Service, Department of Gastroenterology, Rabin Medical Center, Beilinson Campus and Sackler Faculty of Medicine, Tel Aviv University, 39 Jabotinski Street, Petah Tikva, 49100 Israel; The Academic College of Tel Aviv-Jaffa, Tel Aviv, Israel

## Abstract

**Background:**

Rifaximin is a minimally absorbed antibiotic with high luminal activity, used to treat various gastrointestinal diseases. Although rifaximin has been proposed as first line treatment for small intestinal bacterial overgrowth (SIBO), few data are available regarding its efficacy in non-IBS subjects. We aimed to assess the ability of rifaximin to normalize lactulose-H_2_ breath tests in non-IBS subjects with symptoms suggestive of SIBO.

**Materials and methods:**

Consecutive non-IBS patients presenting with bloating and flatulence were prospectively recruited and submitted to lactulose-H_2_ breath testing (LBT). Patients who had a positive result were offered rifaximin 1200 mg daily for 10 days. Breath testing was repeated two weeks after treatment completion in all patients in order to assess for response.

**Results:**

A total of 19 patients with a positive result received rifaximin and repeated the breath test (7 (36.8%) males, age 56.5 ± 17.6 years). The mean peak hydrogen excretion was 13.7 ± 2.8 and 10.3 ± 7.3 ppm at baseline and following rifaximin treatment, respectively (t = 1.98, p = 0.06). LBT normalized in 8/19 (42.1%) subjects. No patients reported symptom resolution. No adverse events were reported.

**Discussion:**

Strengths include the study's prospective design. Limitations include the small sample size and open label design.

**Conclusion:**

Rifaximin was not effective in normalizing LBT in our cohort of non-IBS subjects with symptoms suggestive of SIBO.

## Introduction and background

Small intestinal bacterial overgrowth (SIBO) is a heterogeneous disorder with a clinical presentation ranging from florid malabsorption to minor, non-specific or an absence of symptoms [1]. SIBO is commonly defined as the presence of at least 1 × 10^5^ colony forming units of bacteria per milliliter in a duodenal aspirate [2]. Although classically associated with motility disorders and anatomical abnormalities of the upper gastrointestinal tract, SIBO may be more prevalent than previously thought. This increased prevalence may be due to the availability of noninvasive, inexpensive and easily applicable alternative diagnostic test to direct assay of a duodenal aspirate, such as the lactulose-hydrogen breath test (LBT) [3]. Furthermore, LBT testing allows for serial testing and provides an objective measure of treatment response. Indeed, normalization of the LBT correlates with symptom improvement in IBS patients [4]. Antibiotics are the mainstay of treatment for SIBO. Minimally-absorbed antibiotics, such as rifaximin, offer a theoretical advantage by allowing maximal luminal activity with few systemic adverse effects. Nevertheless, the efficacy of such treatment, as measured by LBT negativity, varies greatly in the reported literature, ranging from 2% to 91% [5,6].

Several studies have assessed rifaximin in patients with SIBO and IBS [7-10]. A recent meta-analysis found that 4-64% of IBS subjects may have a positive diagnostic test suggestive of SIBO. There is controversy whether such IBS patients should be diagnosed as IBS, or rather considered as SIBO, since the symptoms of IBS and SIBO overlap [11]. Given the ambiguity surrounding the etiological role of SIBO in IBS patients, and given the fact that rifaximin may be beneficial for the treatment of IBS even in the absence of SIBO [12], it follows that in order to assess the efficacy of rifaximin for the treatment of SIBO, a homogenous cohort of patients *without* IBS should be used. This is the first study to assess the efficacy of rifaximin in a non-IBS cohort. The rationale for treating SIBO in non-IBS subjects stems from the significant correlation observed between clinical symptoms related to SIBO and abnormal diagnostic tests [13].

### Aim

The aim of this study is to assess the efficacy of rifaximin for the treatment of SIBO in non-IBS subjects, in our geographical region.

## Materials and methods

### Patient eligibility

Between January 2012 and July 2013 consecutive patients over 18 years-old, presenting to the neurogastroenterology clinic at our institution with primary symptoms of bloating and flatulence, were prospectively recruited, and subjected to LBT. None of the subjects complained of chronic recurrent abdominal pain or fulfilled the Rome III criteria for irritable bowel syndrome [14].

Patients who used antibiotics during the previous 6 months, currently used laxatives or promotility agents, or had received bowel preparation for colonoscopy or capsule endoscopy within 30 days, were excluded. Additional exclusion criteria were presence of pancreatic exocrine insufficiency or major concomitant diseases (including active malignancy, hepatic failure and renal insufficiency), hypersensitivity to antibiotics belonging to the rifamycin and/or tetracyclin families, patient unwillingness or inability to provide informed consent, and patient inability to fully complete all phases of the study. Finally, we excluded pregnant and lactating women, as well as premenopausal women not using contraception.

The study was conducted in accordance with the principles of the Declaration of Helsinki and Good Clinical Practice (GCP) and was approved by the Human Subjects Protection Program of Rabin Medical Center.

### Clinical assessment

Prior to LBT, all patients were assessed at a dedicated neurogastroenterology clinic by a study physician (RD) for a thorough clinical assessment and to confirm the absence of IBS. Following LBT patients returned to the study physician for rifaximin prescription and continued to be monitored on an individualized basis. Symptoms were monitored at clinic visits which were scheduled fortnightly.

### Rome III diagnostic questionnaire for irritable bowel syndrome

This self-assessed standardized and validated questionnaire was developed by the Rome Foundation Board to identify functional gastrointestinal disease. For the diagnosis of IBS, patients must have recurrent abdominal pain or discomfort for at least 3 months in the previous 6 months, with 2 or more of the following symptoms: (1) relief with defecation, (2) onset associated with a change in frequency of stool, and (3) onset associated with a change in form (appearance) of stool. All screened subjects completed a validated Hebrew language Rome III Diagnostic Questionnaire for IBS [14]. Only patients that did not fulfill the Rome III criteria for IBS were included and were offered treatment with rifaximin.

### SIBO Evaluation

LBT was performed according to standard protocols [15]. Each subject was submitted to LBT (EC 60 Gastrolyzer 2, Bedfont Scientific, Rochester, UK) following a 3 day low carbohydrate diet, overnight fasting, and chlorhexidine mouthwash. Samples of end-expiratory air were collected before oral administration of 15 g lactulose diluted in 400 mL of water, every 15 min for 90 minutes (15, 30, 45, 60, 75, 90 minutes). The accuracy of the Gastrolyzer was ±2% of reading. The sensor sensitivity was 1 ppm. The test was considered positive for SIBO when an increase over the baseline level was >10 ppm, on the basis of established validation data [16,17].

### SIBO Eradication

Non-IBS patients with a positive LBT received treatment with rifaximin 1200 mg daily for 10 days (Normix, Alfa Wassermann, Italy). Patients were reassessed with LBT two weeks after completion of treatment.

### Statistical analysis

All analyses were performed using SPSS version 21.0 statistical analysis software (IBM Inc, Chicago, IL, USA). Distributions of continuous variables were assessed for normality using the Kolmogorov-Smirnov test (cutoff at p < 0.1). Distributions of all continuous variables deviated significantly from normal so were described as median (min-max) in addition to mean ± standard deviation. Continuous variables were compared by rifaximin treatment exposure using the student's t-test. Nominal variables were described as frequency counts and presented as n(%). Nominal variables were compared using the chi square test. We calculated that at least 22 patients needed to be recruited in order to have a 90% chance of detecting a 50% rate of breath test normalization following treatment with rifaximin. All tests were two-sided and considered significant at p < 0.05.

## Results

### Demographics

**Figure 1:**
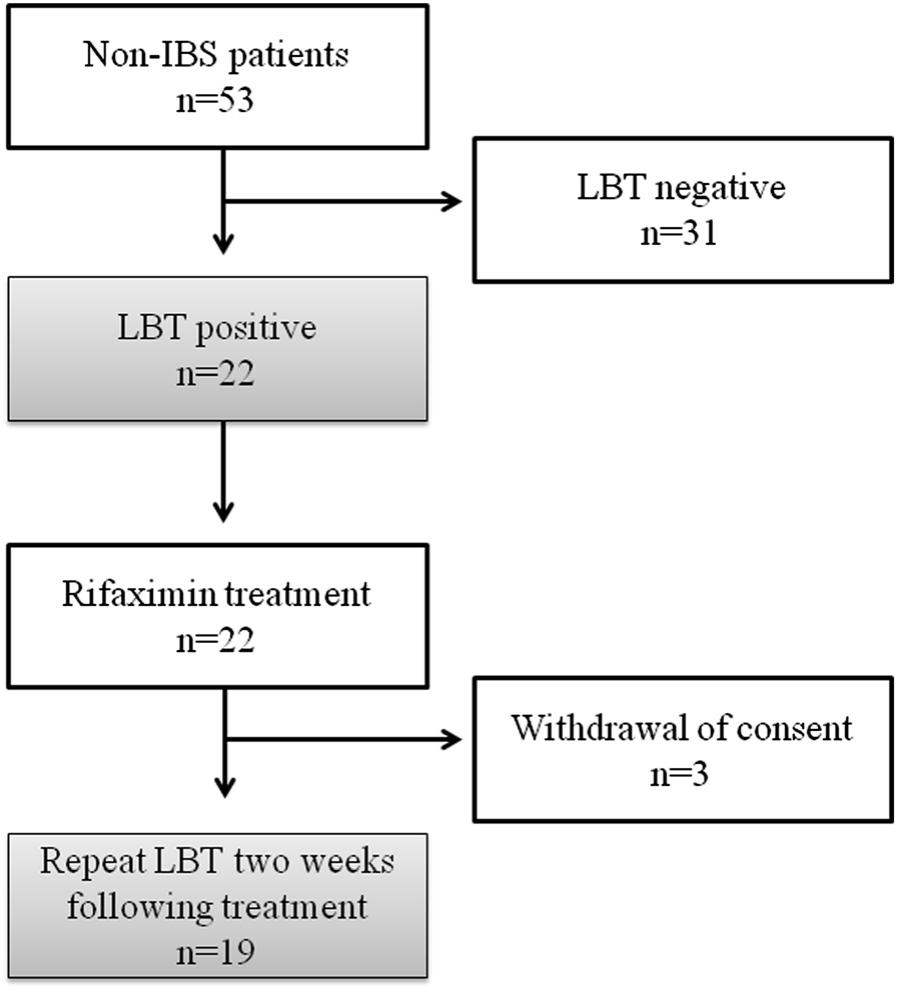
**Study Flowchart.**

A total of 53 non-IBS patients underwent LBT of whom 22 patients (41.5%) had a positive LBT result. All patients were offered primary treatment with rifaximin (1200 mg daily for 10 days). 3 patients who received rifaximin did not undergo follow-up LBT, and therefore 19 patients (7 (36.8%) males, aged 56.5 ± 17.6 years) were included in the final set analysis (Figure 1).

### Clinical characteristics

The clinical characteristics of patients receiving rifaximin for SIBO are shown in Table 1. Among the 19 non-IBS patients receiving rifaximin for treatment of LBT-diagnosed SIBO, 13 (68.4%) received antisecretory therapy, 5 (26.3%) reported diabetes mellitus and 2 patients reported collagen vascular disease.

**Table 1:** **Characteristics of patients receiving rifaximin for small intestine bacterial overgrowth**

	**N (%)**
**Total**	**19 (100)**
**Male**	7(36.8)
**Age, years (SD)**	56.5 (17.6)
**Medications**	
**Anticholinergics**	5 (26.3)
**Calcium channel blocker**	3 (15.8)
**Proton pump inhibitor**	12 (63.2)
**Comorbidities**	
**Diabetes mellitus**	5 (26.3)
**Sjogren’s syndrome**	1 (12.5)
**Penicillin allergy**	3 (15.8)

### SIBO Eradication

**Figure 2:**
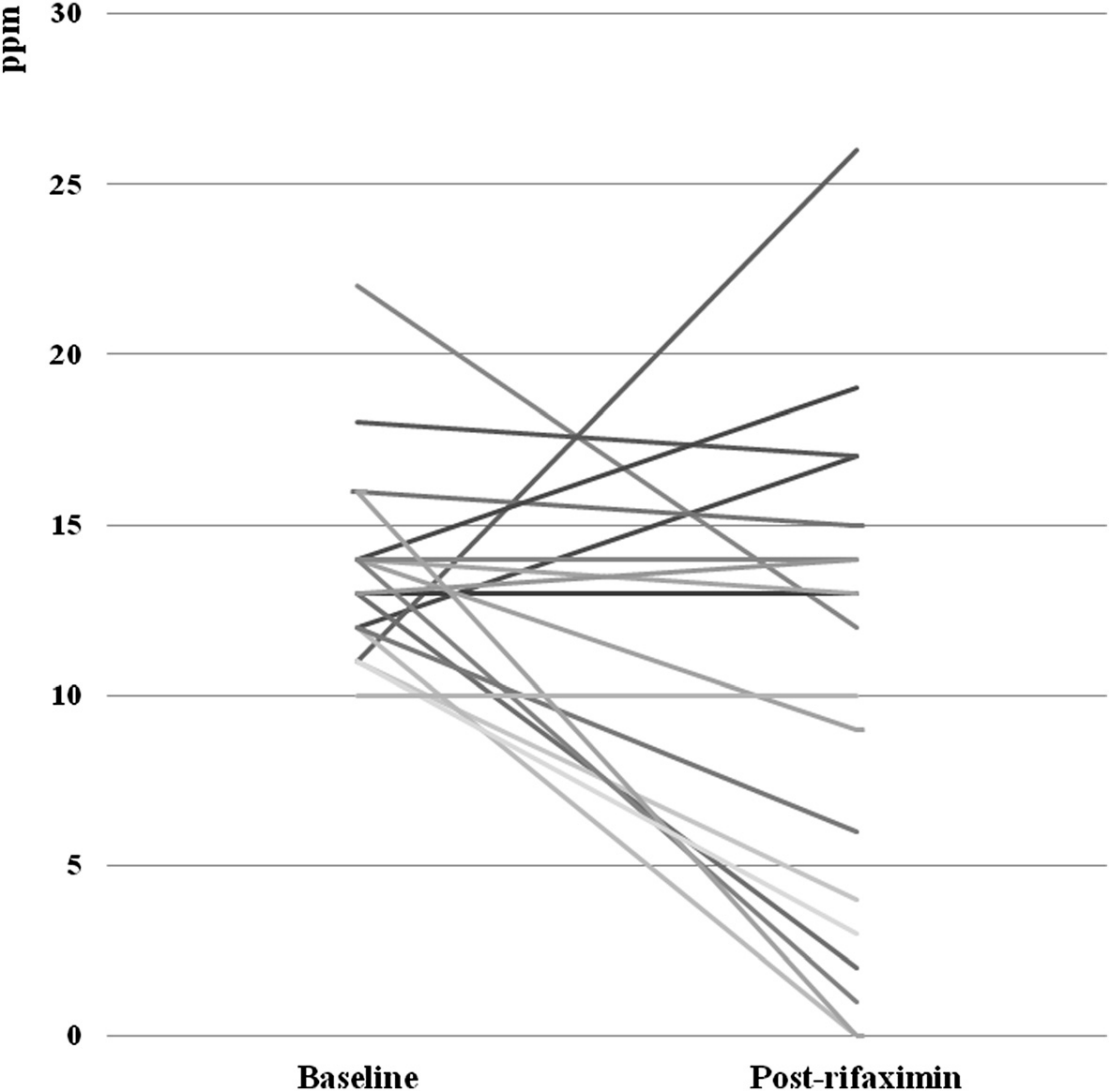
**Peak Hydrogen Excretion at Baseline and Following Rifaxamin.**

The mean peak hydrogen excretion was 13.68 ± 2.83 and 10.26 ± 7.30 ppm at baseline and following rifaximin treatment, respectively (t = 1.98, p = 0.06). Following treatment with rifaximin, LBT normalized in 8/19 (42.1%) subjects, and LBT recordings decreased in 12/19 (63.2%) subjects. In 7/19 (36.8%) subjects the LBT recording increased or remained unchanged (Figure 2). No difference in demographic or clinical characteristics was observed between rifaximin responders and non-responders (Table 2). No patients reported any degree of resolution of either bloating or flatulence. No adverse events were reported.

**Table 2:** **Demographic and clinical features according to rifaximin response**

	**Responders N (%)**	**Non-responders N (%)**	***P*** **value**
**N (%)**	8 (42.1)	11 (57.9)	0.74
**Male**	2 (25)	5 (75)	0.37
**Age, years (SD)**	57.6 (19.5)	55.6 (17.1)	0.72
**Medications**			
**Anticholinergics**	1 (12.5)	4 (36.4)	0.31
**Calcium channel blocker**	1 (12.5)	2 (18.2)	1.00
**Proton pump inhibitor**	5 (62.5)	7 (63.6)	1.00
**Comorbidities**			
**Diabetes mellitus**	3 (37.5)	2 (18.2)	0.61
**Scleroderma**	0 (0)	0 (0)	1.00
**Sjogren’s syndrome**	1 (12.5)	1 (9.1)	1.00

## Discussion

In the present study we describe a prospective cohort of subjects receiving the non-absorbable antibiotic rifaximin for LBT-diagnosed SIBO. Treatment success, measured by normalization of LBT, was only 42%. This is the first such study to emanate from our geographical region.

The dosage of rifaximin 1200 mg and the treatment duration of 10 days was chosen in accordance with previous data demonstrating its superior efficacy compared to lower doses [18,19]. Rifaximin has a broad spectrum of activity, especially against anaerobic intestinal bacteria, including *Bacteroides*, *Lactobacilli* and *Clostridia*, which are frequently responsible for the metabolic derangements in SIBO patients. Its activity profile resembles rifamycin, however toxicity is low since it is minimally absorbed from the gut [20].

Previous studies have assessed the efficacy of rifaximin in IBS patients with symptoms compatible with SIBO. Up to 84% of patients with IBS have a positive LBT, suggesting that the etiology and symptom manifestation of IBS and SIBO may overlap [4]. Pimental, *et al.* in the landmark TARGET trial, randomized IBS patients without constipation to receive rifaximin 1650 mg or placebo for two weeks. Adequate relief of IBS symptoms was reported in 40.8% and 31.2% of treatment and placebo groups, respectively (*p* = 0.01). However, in their study no breath test was performed at baseline or following treatment [21]. Meyrat, *et al.* treated LBT-positive IBS patients with rifaximin, and demonstrated that treatment was associated with significant improvement in symptoms. LBT negativity was 86% following rifaximin treatment, more than twofold the rate we observed in our non-IBS cohort [7]. Several other nonrandomized open labeled studies have reported similar results [8-10]. A recently published meta-analysis concluded that rifaximin is, indeed, more efficacious than placebo for global IBS symptom improvement [OR = 1.57, 95% CI: 1.22–2.01, therapeutic gain 9.8%, number needed to treat (NNT) = 10.2] [12].

We can speculate that the efficacy of rifaximin in IBS may be unrelated to SIBO, since IBS patients with a negative baseline LBT appear to experience symptomatic improvement. This concurs with our experience in non-IBS SIBO patients in whom symptom improvement was absent or minimal, and suggests that rifaximin is effective treatment for symptoms of IBS, but not for the normalization of LBT in patients with bloating and flatulence. Nevertheless, the presence of IBS cannot account for the marked discrepancy in LBT normalization rates between our non-IBS cohort and other IBS cohorts. The most likely explanation is related to geographical, dietary or ethnic differences in microbiome [22].

In addition to IBS, the rate of LBT normalization following rifaximin therapy has been reported in pediatric patients (20-64%) [23,24], patients with ileal Crohn’s disease (100%) [25], microscopic colitis (71%) [26], celiac disease (4%) [27], chronic acid suppression (87%) [6], diabetes (86%) [28], diverticulitis (100%) [29] and systemic sclerosis (73%) [30]. Despite the fact that treatment protocols vary widely between studies, and irrespective of clinical endpoints, the efficacy of rifaximin for the treatment of SIBO assessed by LBT is clearly unpredictable. This fact is corroborated by our study. No studies to date have specifically targeted patients with SIBO in the absence of IBS or any of the aforementioned associated comorbid conditions for rifaximin therapy.

Several studies have examined a potential role for pre- and probiotics in combination with rifaximin for the treatment of suspected SIBO. Normalization of LBT is significantly higher in patients who receive rifaximin in combination with prebiotics, compared to rifaximin alone (87.1% *vs.* 62.1%, p = 0.02) [31]. Sequential treatment of SIBO with rifaximin and probiotics appears more efficacious than rifaximin and prebiotics with respect to symptom reduction [32]. In fact, some centers recommend sequential treatment with rifaximin followed by probiotics as a standard approach to treating SIBO [8].

Our study has several important limitations, including its small size, and open label design. Our power analysis indicated that 22 subjects needed to be recruited, however this was based on the assumption that at least 50% of subjects treated with rifaximin would have a normal LBT at follow-up. In retrospect, 50% was a slight overestimation, as normalization was actually achieved in 42.1% subjects. We did not include a thorough clinical evaluation or screening period, and therefore no conclusions can be drawn regarding the effect of rifaximin on symptoms. Nevertheless, as a laboratory-based study, our results are valid insofar as they demonstrate that rifaximin does not consistently normalize LBT in SIBO patients. A further limitation of our study is related to the ongoing controversy regarding the validity and interpretation of LBT in SIBO. Some clinicians do not regard LBT as the de facto gold standard for the diagnosis of SIBO, and demand indirect supportive evidence including serum vitamin B12 levels and folate levels [16,33]. Inherent problems with LBT include difficulty in distinguishing SIBO from rapid intestinal transit where similar gas production patterns are observed (false positivity) [3]. In fact, it has been suggested that LBT positivity in IBS patients may be related to rapid intestinal transit, and not SIBO [34]. Finally, compliance was not assessed with pill counts or used packages. Nevertheless, due to the high cost of rifaximin treatment in Israel, we presume that compliance was high.

In conclusion, although most subjects with SIBO who were exposed to rifaximin experienced a reduction in peak hydrogen excretion, this reduction lacked statistical significance. Rifaximin eradicated SIBO in fewer than half of treated patients. Larger studies in non-IBS populations are necessary to verify our findings. Further research should consider both clinical and laboratory endpoints, and explore a potential role for pre- and probiotics in the treatment of SIBO.

## References

[1] Dukowicz AC, Lacy BE, Levine GM (2007). Small intestinal bacterial overgrowth: a comprehensive review. Gastroenterol Hepatol (N Y).

[2] American Gastroenterological Association medical position statement (1999). Guidelines for the evaluation and management of chronic diarrhea. Gastroenterology.

[3] Ghoshal UC (2011). How to interpret hydrogen breath tests. J Neurogastroenterol Motil.

[4] Pimentel M, Chow EJ, Lin HC (2003). Normalization of lactulose breath testing correlates with symptom improvement in irritable bowel syndrome. a double-blind, randomized, placebo-controlled study. Am J Gastroenterol.

[5] Jolley J (2011). High-dose rifaximin treatment alleviates global symptoms of irritable bowel syndrome. Clin Exp Gastroenterol.

[6] Lombardo L, Foti M, Ruggia O, Chiecchio A (2010). Increased incidence of small intestinal bacterial overgrowth during proton pump inhibitor therapy. Clin Gastroenterol Hepatol.

[7] Meyrat P, Safroneeva E, Schoepfer AM (2012). Rifaximin treatment for the irritable bowel syndrome with a positive lactulose hydrogen breath test improves symptoms for at least 3 months. Aliment Pharmacol Ther.

[8] Cuoco L, Salvagnini M (2006). Small intestine bacterial overgrowth in irritable bowel syndrome: a retrospective study with rifaximin. Minerva Gastroenterol Dietol.

[9] Majewski M, Reddymasu SC, Sostarich S, Foran P, McCallum RW (2007). Efficacy of rifaximin, a nonabsorbed oral antibiotic, in the treatment of small intestinal bacterial overgrowth. Am J Med Sci.

[10] Peralta S, Cottone C, Doveri T, Almasio PL, Craxi A (2009). Small intestine bacterial overgrowth and irritable bowel syndrome-related symptoms: experience with Rifaximin. World J Gastroenterol.

[11] Ford AC, Spiegel BM, Talley NJ, Moayyedi P (2009). Small intestinal bacterial overgrowth in irritable bowel syndrome: systematic review and meta-analysis. Clin Gastroenterol Hepatol.

[12] Menees SB, Maneerattannaporn M, Kim HM, Chey WD (2012). The efficacy and safety of rifaximin for the irritable bowel syndrome: a systematic review and meta-analysis. Am J Gastroenterol.

[13] Grace E, Shaw C, Whelan K, Andreyev HJ (2013). Review article: small intestinal bacterial overgrowth–prevalence, clinical features, current and developing diagnostic tests, and treatment. Aliment Pharmacol Ther.

[14] Sperber AD, Shvartzman P, Friger M, Fich A (2007). A comparative reappraisal of the Rome II and Rome III diagnostic criteria: are we getting closer to the ???true??? prevalence of irritable bowel syndrome?. Eur J Gastroenterol Hepatol.

[15] Riordan SM, McIver CJ, Walker BM, Duncombe VM, Bolin TD, Thomas MC (1996). The lactulose breath hydrogen test and small intestinal bacterial overgrowth. Am J Gastroenterol.

[16] Gasbarrini A, Lauritano EC, Gabrielli M, Scarpellini E, Lupascu A, Ojetti V, Gasbarrini G (2007). Small intestinal bacterial overgrowth: diagnosis and treatment. Dig Dis.

[17] Lupascu A, Gabrielli M, Lauritano EC, Scarpellini E, Santoliquido A, Cammarota G, Flore R, Tondi P, Pola P, Gasbarrini G, Gasbarrini A (2005). Hydrogen glucose breath test to detect small intestinal bacterial overgrowth: a prevalence case–control study in irritable bowel syndrome. Aliment Pharmacol Ther.

[18] Lauritano EC, Gabrielli M, Lupascu A, Santoliquido A, Nucera G, Scarpellini E, Vincenti F, Cammarota G, Flore R, Pola P, Gasbarrini G, Gasbarrini A (2005). Rifaximin dose-finding study for the treatment of small intestinal bacterial overgrowth. Aliment Pharmacol Ther.

[19] Pimentel M (2009). Review of rifaximin as treatment for SIBO and IBS. Expert Opin Investig Drugs.

[20] Gillis JC, Brogden RN (1995). Rifaximin. A review of its antibacterial activity, pharmacokinetic properties and therapeutic potential in conditions mediated by gastrointestinal bacteria. Drugs.

[21] Pimentel M, Lembo A, Chey WD, Zakko S, Ringel Y, Yu J, Mareya SM, Shaw AL, Bortey E, Forbes WP, TARGET Study Group (2011). Rifaximin therapy for patients with irritable bowel syndrome without constipation. N Engl J Med.

[22] Reischer GH, Ebdon JE, Bauer JM, Schuster N, Ahmed W, Aström J, Blanch AR, Blöschl G, Byamukama D, Coakley T, Ferguson C, Goshu G, Ko G, de Roda Husman AM, Mushi D, Poma R, Pradhan B, Rajal V, Schade MA, Sommer R, Taylor H, Toth EM, Vrajmasu V, Wuertz S, Mach RL, Farnleitner AH (2013). Performance Characteristics of qPCR Assays Targeting Human- and Ruminant-Associated Bacteroidetes for Microbial Source Tracking across Sixteen Countries on Six Continents. Environ Sci Technol.

[23] Scarpellini E, Giorgio V, Gabrielli M, Filoni S, Vitale G, Tortora A, Ojetti V, Gigante G, Fundarò C, Gasbarrini A (2013). Rifaximin treatment for small intestinal bacterial overgrowth in children with irritable bowel syndrome. Eur Rev Med Pharmacol Sci.

[24] Collins BS, Lin HC (2011). Double-blind, placebo-controlled antibiotic treatment study of small intestinal bacterial overgrowth in children with chronic abdominal pain. J Pediatr Gastroenterol Nutr.

[25] Biancone L, Vernia P, Agostini D, Ferrieri A, Pallone F, Biancone L, Vernia P, Agostini D, Ferrieri A, Pallone F (2000). Effect of rifaximin on intestinal bacterial overgrowth in Crohn's disease as assessed by the H2-Glucose Breath Test. Curr Med Res Opin.

[26] Stoicescu A, Andrei M, Becheanu G, Stoicescu M, Nicolaie T, Diculescu M (2012). Microscopic colitis and small intestinal bacterial overgrowth–diagnosis behind the irritable bowel syndrome?. Rev Med Chir Soc Med Nat Iasi.

[27] Chang MS, Minaya MT, Cheng J, Connor BA, Lewis SK, Green PH (2011). Double-blind randomized controlled trial of rifaximin for persistent symptoms in patients with celiac disease. Dig Dis Sci.

[28] Cuoco L, Montalto M, Jorizzo RA, Santarelli L, Arancio F, Cammarota G, Gasbarrini G (2002). Eradication of small intestinal bacterial overgrowth and oro-cecal transit in diabetics. Hepatogastroenterology.

[29] Tursi A, Brandimarte G, Giorgetti GM, Elisei W (2005). Assessment of small intestinal bacterial overgrowth in uncomplicated acute diverticulitis of the colon. World J Gastroenterol.

[30] Parodi A, Sessarego M, Greco A, Bazzica M, Filaci G, Setti M, Savarino E, Indiveri F, Savarino V, Ghio M (2008). Small intestinal bacterial overgrowth in patients suffering from scleroderma: clinical effectiveness of its eradication. Am J Gastroenterol.

[31] Furnari M, Parodi A, Gemignani L, Giannini EG, Marenco S, Savarino E, Assandri L, Fazio V, Bonfanti D, Inferrera S, Savarino V (2010). Clinical trial: the combination of rifaximin with partially hydrolysed guar gum is more effective than rifaximin alone in eradicating small intestinal bacterial overgrowth. Aliment Pharmacol Ther.

[32] Rosania R, Giorgio F, Principi M, Amoruso A, Monno R, Di Leo A, Ierardi E (2013). Effect of probiotic or prebiotic supplementation on antibiotic therapy in the small intestinal bacterial overgrowth: a comparative evaluation. Curr Clin Pharmacol.

[33] Bayeli PF, Mariottini M, Lisi L, Ferrari P, Tedone F (1999). Linee guida sul dismicrobismo intestinale (SIBO: Small Intestinal Bacterial Overgrowth). Minerva Gastroenterol Dietol.

[34] Yu D, Cheeseman F, Vanner S (2011). Combined oro-caecal scintigraphy and lactulose hydrogen breath testing demonstrate that breath testing detects oro-caecal transit, not small intestinal bacterial overgrowth in patients with IBS. Gut.

